# Prevalence and Risk Factors of Mobile Screen Dependence in Arab Women Screened with Psychological Stress: A Cross-Talk with Demographics and Insomnia

**DOI:** 10.3390/jcm14051463

**Published:** 2025-02-21

**Authors:** Omar Gammoh, Abdelrahim Alqudah, Mariam Al-Ameri, Bilal Sayaheen, Mervat Alsous, Deniz Al-Tawalbeh, Mo’en Alnasraween, Batoul Al. Muhaissen, Alaa A. A. Aljabali, Sireen Abdul Rahim Shilbayeh, Esam Qnais

**Affiliations:** 1 Entrepreneurship and Innovation Center, Yarmouk University, Irbid 21163, Jordan; 2 Department of Clinical Pharmacy and Pharmacy Practice, Faculty of Pharmacy, Yarmouk University, Irbid 21163, Jordan; m.alameri@yu.edu.jo (M.A.-A.); mervat.alsous@yu.edu.jo (M.A.); 3 Department of Clinical Pharmacy and Pharmacy Practice, Faculty of Pharmaceutical Sciences, The Hashemite University, Zarqa 13133, Jordan; abdelrahim@hu.edu.jo; 4 E-Learning and Open Education Resources Center, Yarmouk University, Irbid 21163, Jordan; bsayaheen@yu.edu.jo; 5 Department of Translation, Yarmouk University, Irbid 21163, Jordan; 6 Department of Medicinal Chemistry and Pharmacognosy, Faculty of Pharmacy, Yarmouk University, Irbid 21163, Jordan; deniz.altawalbeh@yu.edu.jo; 7 Department of Counseling and Educational Psychology, Faculty of Educational Sciences, Yarmouk University, Irbid 21163, Jordan; moen.na@yu.edu.jo; 8 Department of Modern Languages, Faculty of Arts, Yarmouk University, Irbid 21163, Jordan; batoul@yu.edu.jo; 9 Department of Pharmaceutics and Pharmaceutical Technology, Faculty of Pharmacy, Yarmouk University, Irbid 21163, Jordan; alaaj@yu.edu.jo; 10 Department of Pharmacy Practice, College of Pharmacy, Princess Nourah bint Abdulrahman University, P.O. Box 84428, Riyadh 11671, Saudi Arabia; ssabdulrahim@pnu.edu.sa; 11 Department of Biology and Biotechnology, Faculty of Science, The Hashemite University, Zarqa 13133, Jordan; esamqn@hu.edu.jo

**Keywords:** Jordan, women, mental health, mobile screens

## Abstract

**Background/Objectives**: The current study aims to investigate the rate and the factors associated with mobile screen dependence as a coping mechanism among women residing in Jordan and screened for stress, with a focus on demographics and insomnia. **Methods**: This cross-sectional study with predefined inclusion criteria used validated tools to assess stress, anxiety, and insomnia. **Results**: The data analyzed from 431 women showed that 265 (61.5%) were ≤25 years old, 352 (81.7%) received a university education, and 201 (46.6%) were current students. In addition, 207 (48.0%) reported a dependence on mobile screens for coping, 107 (24.8%) reported severe anxiety, and 180 (41.7%) reported severe insomnia. The multivariable regression analysis revealed that mobile screen dependence—as a personal coping choice—was significantly associated with “students” (OR = 1.75, 95% CI = 1.19–2.57, *p* = 0.004) and “severe insomnia” (OR = 1.07, 95% CI = 1.07–2.32, *p* = 0.02). **Conclusions**: We report that a high rate of mobile dependence is associated with students and insomnia. Prompt action should be taken to raise awareness regarding the proper coping mechanisms in this population.

## 1. Introduction

Screen addiction is a cognitive behavior addiction characterized by excessive phone use and dependence. According to the official website of the Department of Statistics in Jordan, the population of the Hashemite kingdom was estimated to be 11,516,000 at the end of 2023, with females comprising 47.1% of the total. Additionally, Jordan is a youthful country, with one-fifth of its population falling within the youth category, specifically in the age group of 15–24 years, according to a recent report on population estimates prepared by the Department of Statistics in Jordan. In this context, the UNICEF Jordan official website indicates that 63% of Jordan’s population is under the age of 30 [1].

In this technologized era, youths depend on screens—whether smartphones, tablets, or televisions—daily for various purposes, including entertainment, study, and work. The improper excessive use of screens can result in various physical, mental, and psychological/behavioral concerns, including sleep disorders, eye problems, neck and back problems, anxiety, and depression, among many others [2,3,4].

Screens can also serve as a coping mechanism to avoid or manage stressful situations, particularly given the vast amount of news distributed through various social media platforms. Although Jordan has been a safe zone amid armed conflicts for more than a decade, news reports continue to contribute to stress, anxiety, and insomnia among people in the region, including those in Jordan [4,5].

Insomnia is a common sleep disorder characterized by poor sleep quality, latency sleep, and frequent awakening. Insomnia is tightly related to stress, anxiety, and other mental health disorders. Women are more likely to report insomnia [6]. Insomnia and mobile screen dependence are closely related. Evidence from cross-sectional studies in Jordan suggests a bi-directional relationship between them [7].

Women are more vulnerable to experiencing mental health disturbances due to many physiological and environmental stressors, especially in developing countries. This includes hormonal changes, the duties of daily life, and responsibilities including work, study, household work, and others [8]. For example, evidence shows that insomnia and its related problems are higher in women compared to men [6].

Long-term psychological stress is tightly related to mental and behavioral impairments that affect the daily functioning of individuals, especially women [9]. For instance, stress is associated with several somatic immune-related diseases, depression, anxiety, and insomnia [10,11].

Although several investigations have been published about smartphone overuse and mobile screen dependence, to our knowledge, no previous studies have been completely dedicated to investigating this behavior among women screened for stress in Jordan.

The current study aimed to investigate the rate and factors associated with mobile screen dependence as a coping mechanism among women screened for stress in Jordan, with a focus on demographics and insomnia.

## 2. Materials and Methods

### 2.1. Study Design and Settings

The present study followed a cross-sectional design and recruited a sample of Jordanian women according to predefined inclusion criteria through social media platforms related to Yarmouk University. The researchers uploaded the study tool on a Google Form and applied the snowball sampling technique. The objective and steps of the study were all explained to the potential participants before they chose to enroll and approved the consent form. The study obtained approval from Yarmouk University’s IRB committee (number 479). All participants signed an online consent form before enrolling in the study.

### 2.2. Inclusion Criteria

Females aged 18 years old, residing in Jordan, willing to participate, and screened for significant stress using the Perceived Stress Scale (PSS) were included. In brief, the Arabic version of the Perceived Stress Scale, originally developed by Cohen [12], was used. It comprises 14 items designed to measure individual stress over the past month, with a cut-off score of ≥15 reflecting clinically significant stress, as supported by previous research [13].

### 2.3. Exclusion Criteria

Women with a stress score of less than 15 and presenting incomplete or missing data were excluded.

### 2.4. Study Instrument

The study instrument consisted of three distinct sections: demographics, insomnia, and anxiety. The demographics section included structured questions about the participants’ age, marital status, education, employment, and smoking status. This section also included the outcome variable ‘mobile dependence’, through a question identifying stress coping options. The question was formulated as follows: ‘To relieve stress, I depend on “my mobile” or other choices such as sports, eating, and others’. Participants were free to select one or more options.

### 2.5. Anxiety

The severity of anxiety in the sample was assessed using the validated and reliable Arabic version of the General Anxiety Disorder-7 (GAD-7). This self-administered scale consists of 7 questions that assess symptoms of anxiety over the past fourteen days. The scale has a sensitivity of 89% and a specificity of 82% for diagnosing generalized anxiety disorder. For example, questions include ‘Feeling nervous, anxious, or on edge?’, ‘Worrying too much about different things?’, ‘Feeling afraid, as if something awful might happen?’, with response options ‘not at all = 0’ to ‘nearly every day = 3’. Respondents with a score of ≥15 were classified as having severe anxiety symptoms [14,15].

### 2.6. Insomnia

The severity of insomnia was assessed using the translated, validated, and reliable (Cronbach alpha = 0.81) Insomnia Severity Index–Arabic (ISI-A). The ISI-A assesses insomnia severity and its impact on daily life activities over the past 14 days. It consists of seven questions and generates a maximum score of 28. For example, questions include the following: ‘difficulty falling asleep?’, ‘difficulty staying asleep?’, and ‘problems walking up too early?’, with answers ranging from ‘none = 0’, to ‘very = 4”. Participants scoring 15 or above are considered to have severe insomnia [16,17].

### 2.7. Data Analysis

The data presented were categorical and were therefore summarized as frequencies and percentages, as shown in Table 1. To identify which independent variables were associated with mobile screen dependence (dependent variable), a preparatory univariate analysis was conducted (Table 2), followed by a multivariable binary regression analysis, where only significant variables (*p* < 0.05) were retained in the final model. Data were analyzed using SPSS software version 21. The confidence interval was set at 95%, and the significance level was set at *p* < 0.05.

## 3. Results

### 3.1. Study Sample Characteristics

The total number of responders was 612. After applying the inclusion criteria, the number was reduced to 431. The results showed that 265 (61.5%) were ≤25 years old, 267 (61.9%) were single, 352 (81.7%) received a university education, 201 (46.6%) were students, 352 (81.7%) identified themselves as non-smokers, and 377 (87.5) reported no long-term health conditions. In addition, 207 (48.0%) reported depending on mobile screens for coping, while 107 (24.8%) reported severe anxiety and 180 (41.7%) reported severe insomnia (Table 1).

### 3.2. Risk Factors Associated with Mobile Screen Dependence

The preparatory univariate regression analysis (Table 2) revealed the following independent variables as potential confounders: ‘age’, ‘marital status’, ‘employment’, and ‘insomnia’.

These variables were used to create the multivariable binary regression model, as shown in Table 3, which revealed that the dependent variable ‘mobile screens dependence’ was significantly associated with ‘students’ (OR = 1.75, 95% CI= 1.19–2.57, *p* = 0.004) and ‘severe insomnia’ (OR= 1.07, 95% CI = 1.07–2.32, *p* = 0.02).

## 4. Discussion

The current study aimed to investigate the rate and risk factors associated with mobile screen dependence as a coping mechanism among Jordanian women screened for stress. According to our results, almost half of the women reported choosing mobile screens as their primary coping mechanism to relieve stress and anxiety. Additionally, mobile screen dependence among women was significantly associated with being a student and experiencing severe insomnia. To our knowledge, this is the first study to highlight the risk factors associated with mobile screen dependence among women residing in Jordan. Despite the importance of women’s well-being, this topic remains relatively overlooked in many settings.

The study revealed significant correlations between having a dependence on mobile devices and specific variables, such as being a student and having acute insomnia. Students were 1.75 times more likely than non-students to be dependent on mobile screens. This finding is supported by the literature; according to Liu et al., 52.8% of medical students were found to be addicted to smartphones [18]. Another study on smartphone addiction among undergraduates found that 49% of respondents used their phones for at least five hours daily [19]. A recent review explored the reasons for mobile screen addiction among students. In contemporary student life, smartphones serve as platforms for communication and entertainment, in addition to being academic tools. The younger generation extensively uses smartphones for social networking, studying, entertainment, and internet browsing [20].

The use of smartphones as an essential tool is increasing in people’s daily lives. These devices allow people to access information, interact with friends, and manage daily tasks with ease and convenience. Smartphones offer numerous advantages as multi-purpose tools for communication, education, entertainment, and achieving business goals. Despite these advantages and the strong reliance on these devices, increased daily use poses several potential future risks [21].

Some of these risks may affect long-term cognitive functioning, which may hinder students’ ability to maintain focus and engage in deep learning processes. According to a study by Ophir et al. (2009) [22], habitual multitasking—often driven by smartphone technology—can lead to a loss of cognitive control and reduce task management effectiveness. In an increasingly complex and demanding world, these consequences may make hinder children’s academic and professional success [23].

The decline in interpersonal communication skills is another potential risk. The instant communication made possible by smartphones has reduced personal encounters, weakening social ties and emotional intelligence. Furthermore, increased screen use has been linked to decreased psychological well-being, as shown by longitudinal research such as that conducted by Twenge and Campbell (2018) [21]. This raises questions about the state of students’ mental health in a world where digital settings are prevalent. In this context, Livingstone and Smith (2014) pointed out that the misuse of smartphone technology—such as cyberbullying or exposure to harmful content—can have lasting psychological effects [23].

People often check their smartphones in the evening, even during long and stressful work days. However, this behavior can disrupt sleeping habits and patterns, as individuals forgets the time while scrolling through social media pages. In addition, the blue light emitted by smart devices negatively affects the brain’s daily rhythm, tricking it into thinking that it is still early, which can lead to insomnia over time [24].

The continuous use of these devices may harm the eyes and weaken vision. It can also harm the neck and spine. Digital screens emit blue light, which harms eye health and vision. The deterioration of the psychological state of many individuals is accompanied by anxiety or tension as they spend more hours on smartphones, further increasing the risks resulting from this use.

The study also finds a strong correlation between mobile screen dependence and severe insomnia. The likelihood of relying on mobile screens was shown to be higher among those with severe insomnia. These findings align with several previous studies. For example, one study using data from a national health survey of college and university students in Norway found a significant negative correlation between sleep and screen use. These results suggest that students’ screen time significantly impacts both the duration and quality of their sleep. The findings also show a significant correlation between social media addiction and increased rates of sleeplessness [25]. Additionally, several studies have proven that university students who rely heavily on their phones are more likely to experience a poor sleep quality [26,27]. One explanation for this relationship is that exposure to the blue light emitted by screens disturbs sleeping patterns by interfering with melatonin production and circadian rhythms [28].

Moreover, mobile screens can be used by individuals with insomnia as a coping mechanism or to pass time, which exacerbates sleep disturbance [29]. Social media and entertainment content on mobile devices can overstimulate the mind, making it difficult to relax and obtain restful sleep [30]. Thus, being a student with insomnia creates a vicious circle in which an excessive reliance on mobile screens both causes and results in sleep disturbances. To mitigate the detrimental effects of insomnia and mobile screen dependence, it is crucial to address screen time habits and promote improved sleep hygiene, particularly among students.

This work tackles a new and pressing topic regarding women’s mental health and mobile screen dependence during times of armed conflict in the Middle East. The current work provides several strengths, such as the type of the sample and the geographical location (adjacent to zones of armed conflicts). In addition, the current work used a representative sample size, validated measurement tools, and robust data analysis. On the other hand, the cross-sectional design of the study limits our ability to establish causal relationships between variables. Additionally, the self-administered quantitative approach limited accurate diagnosis or qualitative investigation. Another limitation was the lack of information about the content accessed through mobile screens by the respondents. These limitations will be taken into consideration for future research, which will focus on the causes of stress in this population segment, i.e., young women residing in Jordan. This could include further in-depth qualitative studies to unravel any unseen causes of this disabling phenomenon and larger-scale quantitative studies conducted across several geographical locations. Additionally, future studies should address insomnia among women in Jordan. This could involve awareness sessions in universities that highlight the importance of high-quality sleep and the need to consult health care professionals to adequately manage this issue.

## 5. Conclusions

In conclusion, mobile dependency was found to be prevalent among Jordanian women, and was primarily associated with university students and insomnia. The rationale and the beneficial use of technology must be promoted in Jordan to enhance mental health and improve the overall well-being of women during times of stress. The findings of this research pave the way for further qualitative and quantitative studies dedicated to unravelling the causes of stress and related issues, such as insomnia, in this young and fragile population that relies heavily on technology.

## Figures and Tables

**Table 1:** Study sample characteristics (n = 431).

Factor	Category	N (%)
**Age (Years)**	≤25	265 (61.5)
	>25	166 (38.5)
**Marital status**	Single	267 (61.9)
	Married	147 (34.1)
	Divorced	13 (3.0)
	Widow	4 (0.9)
**Education level**	Primary school	2 (0.5)
	Secondary school	33 (7.7)
	University	352 (81.7)
	Postgraduate study	44 (10.2)
**Employment status**	Does not work	111 (25.8)
	Student	201 (46.6)
	Work	105 (24.4)
	Retired	14 (3.2)
**Smoking status**	Non-smoker	352 (81.7)
	Smoker	79 (18.3)
	Use E-cigarette	22 (5.1)
	Use Shisha	56 (13.0)
	Negative smoker	51 (11.8)
**Having chronic diseases**	Yes	54 (12.5)
	No	377 (87.5)
**Screen dependent**	Yes	207 (48.0)
	No	224 (52.0)
**Severe Insomnia**	180 (41.7)	
**Severe Anxiety**	107 (24.8)	

**Table 2:** Univariate analysis of risk factors associated with mobile screen dependence (N = 431).

	Use Screen (N = 207) N (%)	Do Not Use Screens (N = 224) N (%)	*p*-Value
**Age**			0.004 *
≤25	142 (68.6)	123 (54.9)	
>25	65 (31.4)	101 (45.1)	
**Marital status**			0.040 *
Single	140 (67.6)	127 (56.7)	
Married	61 (29.5)	86 (38.4)	
Widow	0 (0.0)	4 (1.8)	
Divorced	6 (2.9)	7 (3.1)	
**Employment status**			0.004 *
Work	42 (20.3)	69 (30.8)	
Does not work	50 (24.2)	55 (24.6)	
Student	112 (54.1)	89 (39.7)	
Retired	3 (1.4)	11 (4.9)	
**Educational level**			0.356
Primary	1 (0.4)	2 (0.4)	
Secondary	12 (5.5)	21 (9.4)	
University level	176 (85.0)	176 (78.6)	
Postgraduate study	18 (8.7)	26 (11.6)	
**Living Place**			0.797
Inside Amman	26 (12.6)	30 (13.4)	
Outside Amman	181 (87.4)	194 (86.6)	
**Smoking**			0.446
Not smoker	166 (80.2)	186 (83.0)	
Smoker	41 (19.8)	38 (17.0)	
**Having chronic diseases**			0.236
Yes	30 (14.5)	24 (10.7)	
No	177 (85.5)	200 (89.3)	
**Having severe insomnia**			0.014 *
Yes	99 (47.8)	81 (36.2)	
No	108 (52.2)	143 (63.8)	
**Having anxiety**			0.211
Yes	57 (27.5)	50 (22.3)	
No	150 (72.5)	174 (77.7)	

* *p*-value < 0.05.

**Table 3:** Binary logistic regression analysis of risk factors associated with mobile screen dependence.

Independent Variable	B	SE	Odds Ratio	95% CI	*p*-Value
**Being a student**	0.558	0.197	1.748	1.19–2.57	0.004 *
**Having severe insomnia**	0.453	0.199	1.572	1.07–2.32	0.023 *

* *p*-value < 0.05. B, regression coefficient; SE, standard error associated with the coefficient B; CI, confidence interval.

## Data Availability

Data will be made available upon reasonable request.

## References

[1] Department of Statistics (2024). Population—Department of Statistics. https://dosweb.dos.gov.jo/population/population-2/.

[2] Wang B., Yao N., Zhou X., Liu J., Lv Z. (2017). The association between attention deficit/hyperactivity disorder and internet addiction: A systematic review and meta-analysis. BMC Psychiatry.

[3] Shi X., Wang A., Zhu Y. (2023). Longitudinal associations among smartphone addiction, loneliness, and depressive symptoms in college students: Disentangling between–and within–person associations. Addict. Behav..

[4] Gammoh O.S., Alqudah A., Qnais E., Alotaibi B.S. (2024). Factors associated with insomnia and fatigue symptoms during the outbreak of Oct. 7th war on Gaza: A study from Jordan. Prev. Med. Rep..

[5] MBlake J., Trinder J.A., Allen N.B. (2018). Mechanisms Underlying the Association Between Insomnia, Anxiety, and Depression in Adolescence: Implications for Behavioral Sleep Interventions.

[6] Zuo L., Chen X., Liu M., Dong S., Chen L., Li G., Zhai Z., Zhou L., Chen H., Wei Y. (2022). Gender differences in the prevalence of and trends in sleep patterns and prescription medications for insomnia among US adults, 2005 to 2018. Sleep Health.

[7] Al Battashi N., Al Omari O., Sawalha M., Al Maktoumi S., Alsuleitini A., Al Qadire M. (2021). The Relationship Between Smartphone Use, Insomnia, Stress, and Anxiety Among University Students: A Cross-Sectional Study. Clin. Nurs. Res..

[8] Lee M.F., Lai C.S. (2020). Mental Health Level and Happiness Index among Female Teachers in Malaysia. Ann. Trop. Med. Public Health.

[9] Alshdaifat E., Absy N., Sindiani A., AlOsta N., Hijazi H., Amarin Z., Alnazly E. (2022). Premenstrual Syndrome and Its Association with Perceived Stress: The Experience of Medical Students in Jordan. Int. J. Women’s Health.

[10] Stojanovich L., Marisavljevich D. (2018). Stress as a trigger of autoimmune disease. J. Neurol. Sci..

[11] Kolahkaj B., Zargar F. (2015). Effect of Mindfulness-Based Stress Reduction on Anxiety, Depression and Stress in Women with Multiple Sclerosis. Nurs. Midwifery Stud..

[12] Cohen S., Kamarck T., Mermelstein R. (1994). Perceived stress scale. Meas. Stress A Guid. Health Soc. Sci..

[13] OGammoh O.S., Al-Smadi A., Alqudah A., Al-Habahbeh S., Weshah F., Ennab W., Al-Shudifat A.E., Bjørk M.H. (2023). The association between fingolimod and mental health outcomes in a cohort of Multiple Sclerosis patients with stress. Eur. Rev. Med. Pharmacol. Sci..

[14] Lo B. (2008). Validation and Standardization of the Generalized Anxiety Disorder Screener (GAD-7) in the General Population. Med. Care.

[15] Gammoh O., Al-Smadi A., Mansour M., Ennab W., Al Hababbeh S., Al-Taani G., Alsous M., Aljabali A.A., Tambuwala M.M. (2023). The relationship between psychiatric symptoms and the use of levetiracetam in people with epilepsy. Int. J. Psychiatry Med..

[16] KSuleiman H., Yates B.C. (2011). Translating the insomnia severity index into Arabic. J. Nurs. Scholarsh..

[17] Morin C. (1993). Insomnia: Psychological Assessment and Management. https://psycnet.apa.org/record/1993-98362-000.

[18] Liu H., Zhou Z., Huang L., Zhu E., Yu L., Zhang M. (2022). Prevalence of smartphone addiction and its effects on subhealth and insomnia: A cross-sectional study among medical students. BMC Psychiatry.

[19] Boumosleh J., Jaalouk D. (2018). Smartphone addiction among university students and its relationship with academic performance. Glob. J. Health Sci..

[20] Zhou B., Mui L.G., Li J., Yang Y., Hu J. (2024). A model for risk factors harms and of smartphone addiction among nursing students: A scoping review. Nurse Educ. Pract..

[21] Twenge J.M., Campbell W.K. (2018). Associations between screen time and lower psychological well-being among children and adolescents: Evidence from a population-based study. Prev. Med. Rep..

[22] Ophir E., Nass C., Wagner A.D. (2009). Cognitive control in media multitaskers. Proc. Natl. Acad. Sci. USA.

[23] Livingstone S., Smith P.K. (2014). Annual research review: Harms experienced by child users of online and mobile technologies: The nature, prevalence and management of sexual and aggressive risks in the digital age. J. Child Psychol. Psychiatry.

[24] Sohn S.Y., Rees P., Wildridge B., Kalk N.J., Carter B. (2019). Prevalence of problematic smartphone usage and associated mental health outcomes amongst children and young people: A systematic review, meta-analysis and GRADE of the evidence. BMC Psychiatry.

[25] Gellis L.A., Lichstein K.L., Scarinci I.C., Durrence H.H., Taylor D.J., Bush A.J., Riedel B.W. (2005). Socioeconomic status and insomnia. J. Abnorm. Psychol..

[26] Ghosh T., Sarkar D., Sarkar K., Dalai C.K., Ghosal A. (2021). A study on smartphone addiction and its effects on sleep quality among nursing students in a municipality town of West Bengal. J. Fam. Med. Prim. Care.

[27] Sohn S.Y., Krasnoff L., Rees P., Kalk N.J., Carter B. (2021). The association between smartphone addiction and sleep: A UK cross-sectional study of young adults. Front. Psychiatry.

[28] Bonmati-Carrion M.A., Arguelles-Prieto R., Martinez-Madrid M.J., Reiter R., Hardeland R., Rol M.A., Madrid J.A. (2014). Protecting the melatonin rhythm through circadian healthy light exposure. Int. J. Mol. Sci..

[29] SBauducco, Pillion M., Bartel K., Reynolds C., Kahn M., Gradisar M. (2024). A bidirectional model of sleep and technology use: A theoretical review of How much, for whom, and which mechanisms. Sleep Med. Rev..

[30] Bengtsson S., Johansson S. (2022). The meanings of social media use in everyday life: Filling empty slots, everyday transformations, and mood management. Soc. Media + Soc..

